# Comparing the predictive values of procalcitonin/albumin ratio and other inflammatory markers in determining COVID-19 severity

**DOI:** 10.12669/pjms.39.2.6856

**Published:** 2023

**Authors:** Tuba Damar Çakırca, Gökhan Çakırca, Ayşe Torun, Ahmet Bindal, Murat Üstünel, Ahmet Kaya

**Affiliations:** 1 Tuba Damar Çakırca, Department of Infectious Diseases and Clinical Microbiology, Sanliurfa Training and Research Hospital, Sanliurfa, Turkey; 2 Gökhan Çakırca, Department of Biochemistry, Sanliurfa Mehmet Akif Inan, Sanliurfa Mehmet Akif Inan Training and Research Hospital, Sanliurfa, Turkey; 3 Ayşe Torun, Department of Infectious Diseases and Clinical Microbiology, Sanliurfa Training and Research Hospital, Sanliurfa, Turkey; 4 Ahmet Bindal, Department of Intensive Care Unit, Sanliurfa Training and Research Hospital, Sanliurfa, Turkey; 5 Murat Üstünel, Department of Biochemistry, Sanliurfa Training and Research Hospital, Sanliurfa, Turkey; 6 Ahmet Kaya, Department of Anesthesiology and Reanimation, Sanliurfa Training and Research Hospital, Sanliurfa, Turkey

**Keywords:** COVID-19 disease severity, Intensive care unit, Neutrophil/lymphocyte ratio, Procalcitonin/albumin ratio

## Abstract

**Objective:**

To examine the relationship between COVID-19 severity and procalcitonin/albumin ratio (PAR) and compare the PAR with oft-reported inflammatory markers, including procalcitonin, white blood cell (WBC), neutrophil/lymphocyte ratio (NLR) and C-reactive protein (CRP).

**Methods:**

In this retrospective research study conducted at Sanliurfa Training and Research Hospital during May to September 2020; total, 577 adult subjects diagnosed with COVID-19 were included and categorized into two groups based on place of hospitalization: the intensive care unit (ICU) group (n=151) and the general ward (GW) group (n=426). Laboratory test results and demographic characteristics of the subjects were recorded.

**Results:**

PAR, NLR, CRP, WBC, neutrophil and procalcitonin values were markedly higher in the ICU group than in the GW group. On the contrary, lymphocyte count and albumin level were markedly lower. PAR showed positive correlations with WBC, NLR, and CRP. Multivariate analysis showed that advanced age, presence of hypertension, elevated PAR, WBC, NLR, urea and lactate dehydrogenase levels were independent risk factors associated with the need for intensive care in COVID-19 subjects. Among them, the PAR showed the highest odds ratio (5.564) for ICU admission. Additionally, the area under the ROC curve of the PAR (0.888) was markedly greater than that of WBC (0.777), NLR (0.822), CRP (0.842) and procalcitonin (0.870).

**Conclusions:**

This study revealed that PAR was superior to procalcitonin, WBC, NLR and CRP in determining COVID-19 severity. PAR was an important predictor of ICU requirement in COVID-19 cases.

## INTRODUCTION

The COVID-19 disease, which originated in Wuhan, China in the last quarter of 2019, continues to pose a major threat to public health.^1^ The number of patients and deaths due to COVID-19 is still increasing all over the world.^2^ COVID-19 patients are classified into four groups according to the level of disease severity as mild, moderate, severe and critical. Patients with mild/moderate COVID-19 disease have a good prognosis, but some of these patients may progress rapidly to severe or critical illness, which are difficult to treat and have poor clinical outcomes.^3^ A report showed that 61.5% of critically ill COVID-19 patients died within 28 days.^4^ Hence, early recognition and timely initiation of appropriate treatment for critically ill patients may be of great importance in improving the prognosis.

The roles of biochemical, hematological and inflammatory biomarkers in evaluating the course and outcome of COVID-19 have been the focus of research. Many studies have reported changes in common laboratory parameters in patients suffering from severe COVID-19 infection, including an increase in procalcitonin levels and a decrease in albumin levels.^5^-^8^ Moreover, procalcitonin and albumin tests have been found to be strongly related to progression and prognosis of COVID-19.^9^,^10^

The procalcitonin to albumin ratio (PAR) is a new inflammatory index determined by dividing procalcitonin level to albumin level, and recent studies have reported that the PAR is a reliable indicator for the diagnosis and severity estimation of infectious diseases such as urosepsis and nosocomial bloodstream infection.^11^,^12^ However, until now, there is no study investigating the usefulness of the PAR in the assessment of COVID-19 severity. In the present research, our aim was to investigate the power of PAR in determining the severity of COVID-19 infection. In addition, we compared the PAR with common inflammatory markers including procalcitonin, white blood cell (WBC), neutrophil/lymphocyte ratio (NLR) and C-reactive protein (CRP).

## METHODS

This retrospective research was conducted on 577 patients aged over 18 years infected with COVID-19 in Sanliurfa Training and Research Hospital, from May to September 2020.

### Inclusion and Exclusion Criteria:

All patients included in the study had positive COVID-19 PCR test results and were separated into two categories as intensive care unit (ICU) group (n=151) and general ward (GW) group (n=426) based on place of hospitalization. Patients in the ICU group met at least one of the following criteria, according to Chinese guidelines:^13^ (a) respiratory failure needing ventilator support; (b) Shock; (c) Other organ failure requiring ICU follow-up. *Exclusion criteria* included negative PCR test for SARS-CoV-2; pregnancy; malignancy; chronic liver disease; chronic kidney disease; hematological disorder; missing laboratory data; and COVID 19 patients discharged from the emergency department.

The present single-center retrospective research was approved by the Turkish Ministry of Health and Harran University Ethics Committee (approval no. HRU/21.14.08). The basic characteristics, underlying comorbidities and routine laboratory test results at admission of COVID-19 cases were obtained from the hospital information system. Urea, creatinine, albumin, liver transaminases, bilirubin, lactate dehydrogenase (LDH), CRP and procalcitonin levels were detected in Cobas 8000 device (Roche, Germany), while complete blood count parameters were detected in Sysmex XN-1000 device (Sysmex, Japan). Moreover, procalcitonin/albumin ratio (PAR) and neutrophil/lymphocyte ratio (NLR) of all participants were calculated.

### Statistical analysis:

SPSS (version 21.0) software was used for all analyses. Kolmogorov–Smirnov test was used to assess normality. Comparison of variables was done with Mann-Whitney u-test, Student t-test, chi-square test, or Fisher’s exact test. Correlations between PAR and procalcitonin, WBC, NLR and CRP were made with Spearman correlation test. To identify independent predictors of ICU admission, multivariate regression analysis with the backward stepwise (wald) method was performed after univariate analysis. Possible factors with a p value less than 0.20 on univariate analysis were included in multivariate analysis. The predictability of the variables in determining disease severity was evaluated by ROC analysis. The area under the curve (AUC) values of the variables were compared with the DeLong test.

## RESULTS

Demographic data, comorbidities and laboratory test results for the ICU (n=151) and GW (n=426) groups are presented in Table-I. Age, male gender as well as the frequency of hypertension, cardiovascular disease, dyslipidemia and cerebrovascular disease comorbidities were found to be markedly higher in the ICU group than in the GW group (All p<0.05). ICU patients had higher WBC, neutrophil count, urea, creatinine, aspartate transaminase (AST), total bilirubin, LDH, CRP, procalcitonin, PAR and NLR values but lower lymphocyte count and albumin level than those with GW patients. (All p<0.05). No difference was seen in ICU and GW patients in terms of hemoglobin, thrombocyte and alanine transaminase (ALT) levels (All p>0.05). As seen in Table-II, there was a low positive correlation between PAR and WBC, while there was a moderate positive correlation between PAR and NLR as well as PAR and CRP. We also determined the risk factors for admission to ICU by uni- and multivariate logistic regression analyses. Variables that showed statistical significance in the baseline comparison were selected for logistic regression analysis. Univariate analysis identified age, male sex, hypertension, cardiovascular disease, dyslipidemia, cerebrovascular disease, urea, creatinine, AST, total bilirubin, LDH, albumin, procalcitonin, WBC, CRP, NLR and PAR as possible risk factors for ICU admission. Procalcitonin and albumin were not included in the multivariate logistic model due to the multicollinearity problem with PAR. According to multivariate analysis, age>57 years (Odds ratio [OR] 2.719, p=0.005), presence of hypertension (OR 1.921, p=0.044), WBC>7.94 x10^3^/µL (OR 2.056, p=0.033), urea >43.2 mg/dL (OR 2.637, p=0.003), LDH>360 U/L (OR 4.740, p<0.001), NLR>4.75 (OR 2.291, p=0.018) and PAR>0.03 (OR 5.564, p<0.001) were detected as independent factors (Table-III).

**Table-I:** Demographic characteristics and laboratory findings of COVID-19 patients on admission.

	GW group (n=426)	ICU group (n=151)	P value
Age, years	50 (39-64)	70 (59-77)	<0.001
Gender (male), n (%)	198 (46.5)	91 (60.3)	0.004
***Coexisting disorders***			
Diabetes mellitus, n (%)	93 (21.8)	42 (27.8)	0.136
Hypertension, n (%)	119 (27.9)	82 (54.3)	<0.001
Cardiovascular disease, n (%)	45 (10.6)	44 (29.1)	<0.001
Dyslipidemia, n (%)	56 (13.1)	43 (28.5)	<0.001
Respiratory system disease, n (%)	67 (15.7)	34 (22.5)	0.059
Cerebrovascular disease, n (%)	8 (1.9)	10 (6.6)	0.011
***Laboratory data***			
WBC, x10^3^/µL	6.02 (4.84-7.64)	9.82 (7.3-14.43)	<0.001
Neutrophil, x10^3^/µL	3.82 (2.82-5.35)	8.28 (5.18-11.84)	<0.001
Lymphocyte, x10^3^/µL	1.51 (1.14-1.94)	1.11 (0.75-1.57)	<0.001
Hemoglobin, g/dl	13.5±1.64	13.4 ±1.8	0.452
Platelet, x10^3^/µL	226 (184-269)	224 (175-304)	0.537
Urea, mg/dL	30.6 (24.1-38.5)	53.4 (36.9-77.4)	<0.001
Creatinine, mg/dL	0.94 (0.79-1.1)	1.13 (0.94-1.53)	<0.001
Alanine transaminase, U/L	23.8 (16.4-36.2)	26.8 (16.6-40.8)	0.218
Aspartate transaminase, U/L	28.9 (21.6-38.7)	39.2 (28.6-59.7)	<0.001
Total bilirubin, mg/dL	0.35 (0.23-0.5)	0.47 (0.34-0.69)	<0.001
LDH, U/L	270 (221-330)	438 (313-564)	<0.001
CRP, mg/L	15.6 (5.1-44.3)	92.9 (44.4-158.5)	<0.001
Albumin, g/dL	4.27 (3.99-4.53)	3.53 (3.23-3.87)	<0.001
Procalcitonin, ng/mL	0.06 (0.04-0.09)	0.21 (0.11-0.60)	<0.001
NLR	2.46 (1.72-3.76)	6.83 (4.04-13.31)	<0.001
PAR	0.015 (0.01-0.021)	0.063 (0.031-0.168)	<0.001

The data were presented as median (25-75 percentile), mean±standard deviation or numbers (%) as appropriate. GW: General ward, ICU: intensive care unit,WBC: White blood cell count, LDH: Lactate dehydrogenase, CRP: C-reactive protein, NLR: Neutrophil/lymphocyte ratio, PAR: procalcitonin/ albumin ratio.

**Table-II:** Correlations of PAR with common inflammatory markers in COVID-19 patients.

		WBC	NLR	CRP
PAR	r	0.380	0.558	0.667
	p	<0.001	<0.001	<0.001

WBC: White blood cell count, CRP: C-reactive protein, NLR: Neutrophil/lymphocyte ratio.

**Table-III:** Multivariate logistic regression analysis of risk factors associated with ICU admission.

Variables	Odds ratio	95% Confidence Interval		P value

		Lower bound	Upper bound	
Age (>57years)	2.719	1.352	5.467	0.005
Hypertension	1.921	1.019	3.622	0.044
WBC (>7.94 x10^3^/µL)	2.056	1.059	3.992	0.033
Urea (>43.2 mg/dL)	2.637	1.387	5.014	0.003
LDH (>360 U/L)	4.740	2.569	8.746	<0.001
NLR (>4.75)	2.291	1.153	4.551	0.018
PAR (>0.03)	5.564	2.920	10.599	<0.001

WBC: White blood cell count, LDH: Lactate dehydrogenase, NLR: Neutrophil/lymphocyte ratio, PAR: procalcitonin/albumin ratio.

**Fig.1:**
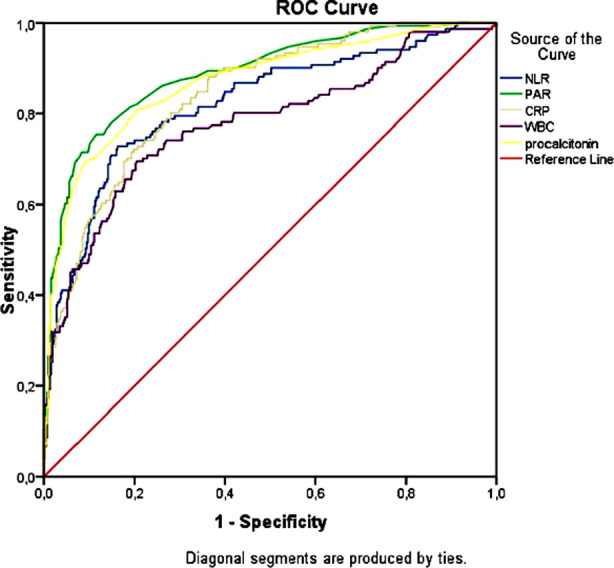
ROC curves of PAR, procalcitonin, WBC, NLR and CRP to estimate COVID-19 Patients requiring ICU care.

ROC analysis results of PAR and other inflammatory markers are summarized in Table-IV and Fig 1. PAR above than 0.03 value showed a good accuracy to identify ICU patients with AUC=0.888, the high specificity (88.5%) and sensitivity (75.5%). Pairwise comparison results exhibited that the AUC value of PAR (0.888) was markedly higher than that of WBC (0.777), NLR (0.822), CRP (0.842) and procalcitonin (0.870) (All p<0.05).

**Table-IV:** ROC analysis results of PAR and other inflammatory markers to predict severity of COVID-19.

	AUC (95% CI)	Cut-off	Sensitivity (%)	Specificity (%)	P value	P value*
PAR	0.888 (0.859-0.912)	0.03	75.5	88.5	<0.001	_
Procalcitonin	0.870 (0.840-0.897)	0.13	69.5	90.8	<0.001	<0.001
WBC	0.777 (0.741-0.811)	7.94	69.5	79.6	<0.001	<0.001
NLR	0.822 (0.789-0.853)	4.75	72.9	83.8	<0.001	0.003
CRP	0.842 (0.810-0.871)	52.1	72.2	80.3	<0.001	0.019

PAR: procalcitonin/albumin ratio, WBC: White blood cell count, NLR: Neutrophil/lymphocyte ratio, CRP: C reactive protein.

* P values obtained by pairwise comparison of AUC values between PAR and procalcitonin, NLR and CRP

## DISCUSSION

This research is the first to evaluate the relationship between COVID-19 severity and PAR. We found that PAR was superior to procalcitonin, WBC, NLR, and CRP in the early recognition of COVID-19 patients requiring ICU care. Additionally, PAR was an important predictor of ICU requirement in these patients.

Laboratory markers are of great importance in detecting the severity and prognosis of COVID-19 disease for early interventions. Studies have reported significant laboratory abnormalities such as lymphopenia, neutrophilia, hypoalbuminemia, and elevated CRP, procalcitonin and D-dimer levels were seen in patients infected with severe COVID-19,^5^-^8^ which is consistent with our findings.

Procalcitonin, the precursor of the calcitonin, is an important inflammatory marker that can be used to identify bacterial infections and guide antibacterial therapy. The level of this marker may also be elevated in non-infectious conditions like trauma, surgery, burns, and rhabdomyolysis.^14^ In case of bacterial infection, procalcitonin production is induced directly by bacterial endotoxins and lipopolysaccharides or indirectly by inflammatory cytokines such as interleukin-six.^15^ On the contrary, procalcitonin production is inhibited by increased interferon-gamma in viral infections,^16^ which explains why procalcitonin levels remain within the reference range in patients with uncomplicated COVID-19 infection and increased procalcitonin levels in severe COVID-19 patients may indicate concomitant bacterial infections.^17^ A meta-analysis also showed that increased procalcitonin levels in COVID-19 patients may be due to bacterial coinfections and/or enhanced release of some cytokines especially interleukin-six.^18^ Previous reports have demonstrated that procalcitonin levels are higher in severe COVID-19 patients than in non-severe COVID-19 patients.^5^,^6^,^10^,^19^ Similarly, we detected that ICU patients had higher procalcitonin levels than GW patients, suggesting that procalcitonin may be associated with the disease severity. Kokturk et al. also noted that procalcitonin was an independent risk factor with the highest odds ratio compared to other laboratory factors for mortality in COVID-19 cases.^20^

Albumin is one of the negative acute phase reactants produced by the liver and has functions such as maintenance of colloid osmotic pressure, protection of microvascular integrity, intravascular transport of various molecules, and antioxidant defenses.^21^ In addition, serum albumin is a sensitive indicator of nutritional status, and a decrease in the levels of this protein has been identified as a poor prognosis in various diseases.^22^,^23^ Huang et al. demonstrated that COVID-19 patients have significantly low albumin levels, which is thought to be due to liver damage caused by the COVID-19-induced cytokine storm.^24^ Bassoli et al. observed a gradual decrease in albumin levels with increasing disease severity in COVID-19,^25^ which is in agreement with our study results since the amount of serum albumin at admission to the hospital was markedly lower in the ICU group than in the GW group. A meta-analysis of 67 studies involving 19,760 COVID-19 patients emphasized that decreases in albumin levels were associated with both disease severity and worse outcomes in these patients.^9^

Based on this information, we consider that the combination of procalcitonin and albumin parameters, which reflect both inflammatory and nutritional status, may increase the estimation of COVID-19 disease severity. The PAR index, which is the ratio of procalcitonin to albumin, has previously been proposed as a potential marker for the diagnosis and severity estimation of infectious diseases.^11^,^12^ Luo et al. demonstrated a markedly elevated PAR in patients with uroseptic shock compared with those urosepsis without shock and speculated that this index may indicate the severity of urosepsis. They also found that the PAR was better at predicting urosepsis than traditional inflammatory biomarkers such as CRP and leukocytes.^11^ Deng et al. reported that the albumin/procalcitonin ratio is a potential marker for early detection and severity of nosocomial bloodstream infection among patients with intracerebral hemorrhage.^12^ Our results indicated that PAR was markedly higher in the ICU patients compared to GW patients, and PAR was positively correlated with WBC, NLR, and CRP levels. In multivariate analysis, advanced age, presence of hypertension, elevated PAR, WBC, NLR, urea and LDH were independent factors for ICU requirement in our study. Among them, the PAR showed the highest odds ratio. Furthermore, ROC analysis revealed that the AUC of the PAR was statistically greater than that of the procalcitonin, WBC, NLR and CRP, indicating that the PAR was superior to these markers in detecting COVID-19 patients requiring ICU support.

### Strengths of the study:

It includes a large sample size of 577 patients infected with COVID-19 and the first study to examine the usefulness of PAR in identifying patients needing ICU support.

### Limitations of the study:

First, this study was designed retrospectively. In this context, exclusion of patients with missing procalcitonin or albumin values from the study may have created selection bias. Second, generalization of our results may be limited as the data were obtained from a single center. Third, there was no information on whether the patients were taking certain medications, such as antibiotics, that could affect procalcitonin levels before admission to the hospital. Fourth, there was insufficient data on how many patients in each group had bacterial co-infection. Finally, regular monitoring and comparison of the PAR during the disease course could not be made.

## CONCLUSION

Our data revealed that PAR is potentially better associated with COVID-19 disease severity than common inflammatory markers such as procalcitonin, WBC, NLR, and CRP, and is a strong independent risk factor for ICU admission. Accordingly, PAR can assist clinicians in identifying high-risk COVID-19 patients, thereby significantly reducing length of hospital stay, incidence of critical illness, and in-hospital death rates.

### Authors Contribution:

**TDC:** Conception & design, analysis, writing, revision of the article and final approval.

**GC:** Literature search, analysis, writing, revision of the article and final approval. He is also responsible for the integrity and accuracy of the study.

**AT:** Data collection, analysis, revision of the article and final approval.

**AB, MU & AK:** Data collection, SPSS data uploading, revision of the article and final approval.
